# Vitamin D Receptor in Muscle Atrophy of Elderly Patients: A Key Element of Osteoporosis-Sarcopenia Connection

**DOI:** 10.14336/AD.2018.0215

**Published:** 2018-12-04

**Authors:** Manuel Scimeca, Federica Centofanti, Monica Celi, Elena Gasbarra, Giuseppe Novelli, Annalisa Botta, Umberto Tarantino

**Affiliations:** ^1^Department of Biomedicine and Prevention, University of Rome “Tor Vergata”, Via Montpellier 1, Rome 00133, Italy.; ^2^IRCCS San Raffaele, 00166, Rome, Italy.; ^3^Department of Orthopedics and Traumatology, "Tor Vergata" University of Rome, "Policlinico Tor Vergata" Foundation, Rome, Italy.; ^4^Neuromed IRCCS, Pozzilli (IS), Italy.; ^5^Department of Experimental Medicine and Surgery, University “Tor Vergata”, Rome 00133, Italy.

**Keywords:** Vitamin d receptor, polymorphisms, sarcopenia, osteoporosis, osteoarthritis, aging

## Abstract

In this study, we investigated the relationship between sarcopenia (evaluated in term of fibers atrophy), vitamin d receptor protein expression and *TaqI/Cdx2/FokI* VDR genotypes in an Italian cohort of osteoporosis(n=44) and osteoarthritis (n=55) patients. Muscle biopsies were fixed and investigated by both immunohistochemistry (vitamin d receptor expression) and transmission electron microscopy (satellite stem cells niches). Vitamin d receptor polymorphisms were studied on DNA extracted from muscle paraffin sections. For the first time, we reported that aging differently affects the VDR activation in OA and OP patients. In particular, while in OP patients we observed a significant reduction of VDR positive myonuclei with age, no “age effect” was observed in OA patients. The frequent activation of VDR could explain the lower number of atrophic fiber that we observed in OA patients respect to OP. From genetic point of view, we showed a putative association among polymorphisms *FokI* and *Cdx2* of VDR gene, vitamin d receptor activation and the occurrence of sarcopenia. Altogether these data open new prospective for the prevention and cure of age-related muscle disorders.

The loss of muscle mass (often referred to as sarcopenia) is one of the most relevant changes occurring in aging. Sarcopenia and the associated reduced muscle strength result in reduced mobility, loss of independence and increased risk of fractures [1]. Muscle wasting is due to myofiber atrophy and loss caused by increased inflammation leading to proteosomal degradation, apoptosis, altered autophagy and decreased myogenic potential [2,4]. In addition, profound metabolic changes occur in myofibers during aging, included increased mitochondrial dysfunctions, which contribute altering cell anabolism and reducing contractile force generation, thus leading to loss of muscle mass and strength [5]. Skeletal muscle is in close relationship with bone tissue [1, 6-7] and emerging evidence suggests that vitamin D may play a direct role in both muscle and bone homeostasis [8,9]. Numerous clinical studies demonstrated the association between vitamin D serum level, 25-hydroxyvitamin D (25(OH)D), and muscle mass and strength [10,11]. In addition, case control and cohort studies have suggested that high (25(OH)D) levels were associated with a reduced of falls and fractures in the senior population [12]. *In vitro* experiment demonstrated that vitamin D is able to induce calcium influx, intracellular signaling and gene expression in muscle stem cells [13,14]. One of the most widely studied candidate genes for sarcopenia is Vitamin D receptor (VDR), due to its key regulatory role in calcium homeostasis and skeletal muscle function [15]. Endo et al. found that the muscle fibers of VDR null mice were smaller and had persistently increased expression of early markers of myogenic differentiation compared with controls [16]. The identification of the VDR on muscle cells provided further support for a direct effect of vitamin D on muscle tissue [17]. In this context, for the first-time Bischoff-Ferrari et al. reported the expression of VDR in skeletal muscle, demonstrating that older age was significantly associated with decreased of nuclear expression of VDR, independent of biopsy location [18]. Nevertheless, the molecular mechanism in which vitamin D regulate musculoskeletal function by VDR activation is longstanding. The VDR is a nuclear receptor of 50 kDa, belonging to class 2 of the family of steroid receptors. The *VDR* gene is located on chromosome 12q.13.1 and contains nine exons [19-21]. The *VDR* gene has several polymorphic sites that have been examined in relation to skeletal muscle traits such as *FokI*, *BsmI*, *TaqI* and *Cdx2*. The *BsmI* and *TaqI* variants are in *linkage disequilibrium* and significant haplotype associations with skeletal muscle strength have been observed [22,23].

The *FokI* polymorphism affects the translational start site of *VDR*. Several studies reported that homozygotes for the C allele showed a two-fold risk to develop sarcopenia compared to T/T homozygotes and C/T heterozygotes [23,24]. Another commonly studied *VDR* polymorphism is *Cdx2* polymorphism, which is a functional binding site for the intestinal specific transcription factor *Cdx2* in the promoter region of the *VDR* gene. *Cdx2* polymorphisms have been associated with increased BMD and decreased risk of fracture in Caucasian and Japanese populations [25-28]. Recently, Ling et al. (2016) examined the relationship between *Cdx2* polymorphism and serum 25(OH)D levels, bone mineral density (BMD) and fracture in Chinese population [29,30]. They found that the A allele in *Cdx2* polymorphism in the *VDR* gene was associated with serum 25(OH)D levels, increased BMD and decreased risk of fracture in women [29,30].

In this study, we investigated the relationship between sarcopenia (evaluated in term of fibers atrophy), VDR protein expression and *TaqI*/*Cdx2*/*FokI VDR* genotypes in an Italian cohort of osteoporosis(n=44) and osteoarthritis (n=55) patients.

## MATERILAS AND METHODS

### Patients

We enrolled 99 patients who underwent hip surgery in the Orthopedic Department of “Tor Vergata” University Hospital in the period June 2013-February 2016. Specifically, we enrolled 44 consecutive patients who underwent hip arthroplasty for subcapital fractures of the femur (39 women and 5 men), and 55 consecutive patients who underwent hip arthroplasty for osteoarthritis (OA) (40 women and 15 men). Exclusion criteria were history of cancer, myopathies or other neuromuscular diseases or chronic administration of corticosteroid for autoimmune diseases (more than 1 month), diabetes, alcohol abuse, and Hepatitis B virus, Human Immunodeficiency Virus or Hepatitis C virus infections. All experiments described in the present study were approved by the ethics committee of “Policlinico Tor Vergata” (approval reference number# 85/12). All experimental procedures were carried out according to The Code of Ethics of the World Medical Association (Declaration of Helsinki). Informed consent was obtained from all patients prior to surgery. Specimens were handled and carried out in accordance with the approved guidelines.

### Bone mineral density evaluation 

DXA was performed with a Lunar DXA apparatus (GE Healthcare, Madison, WI, USA). Lumbar spine (L1-L4) and femoral (neck and total) scans were performed, and BMD was measured according to manufacture recommendations [31]. Dual-energy X-ray absorption-metry (DXA) measures BMD (in grams per square centimeter), with a coefficient of variation of 0.7 %. For patients with fragility fractures, BMD was measured on the uninjured limb. For OA patients, measurements were performed on the non-dominant side, with the participants supine on an examination table with their limbs slightly abducted [32]. DXA exam was performed one day before surgery for OA patients, and one months after surgery for osteoporotic (OP). The results were expressed as T-scores.

### Radiological evaluation

Hip x-rays were performed in order to check the fracture or to assessed hip OA. Kellgren-Lawrence scale (K?-?L) was used in order to determine the severity of osteoarthritis. The Kellgren and Lawrence system is a method of classifying the severity of OA using five grades. This classification was proposed by Kellgren et al. in 1957 [33]. It includes: grade 0 if no radiographic features of OA are present; grade 1 if doubtful joint space narrowing (JSN) and possible osteophytic lipping; grade 2 if definite osteophytes and possible JSN on anteroposterior weight-bearing radiograph; grade 3 if multiple osteophytes, definite JSN, sclerosis, possible bony deformity; grade 4 if large osteophytes, marked JSN, severe sclerosis and definite bony deformity. Two orthopaedists independently assessed all radiographs. Patients with a grade of K?-?L?≥?2 were considered osteoarthritic.

### Hematochemical exam 

Concentration assays of (25(OH)D) was quantified by routine clinical laboratory methods (Vista, Centaur and Immulite Siemens Healthcare Diagnostic, Milano, Italy).

### Sampling

During open surgery for hip arthroplasty, muscle biopsies were taken from the upper portion of the vastus lateralis. Sample withdrawals were performed for histological analysis excluding macroscopic alteration of skeletal muscle biopsy as necrosis areas.

### Histology

Muscle biopsies were fixed in 4% paraformaldehyde for 24 hours and paraffin embedded. Three-micrometer thick sections were stained with hematoxylin and eosin (H&E) and the histological evaluation blindly was performed by two pathologists.

### Morphometric Analysis

In order to assess fibers atrophy, a minimum of 250 muscle fibers per biopsy have been evaluated, comparing minimum transverse diameter and cross-sectional area of type I and type II fibers for relative prevalence. A threshold diameter lower than 30?μm characterized atrophic fibers. In addition, we reported data concerning the percentage the atrophic fibers <30 μm, 30 μm <x> 50 μm, and > 50 μm.

### Immunohistochemistry

Slow myosin, fast myosin, Paired box protein-7 (PAX7) and VDR expression were assed in muscle biopsies by immunohistochemistry. Briefly, antigen retrieval was performed on 4-μm-thick paraffin sections using EDTA citrate pH 7.8 or Citrate pH 6.0 buffers for 30 min at 95 °C. Sections were then incubated for 1 hour at room temperature with primary antibodies (listed in Table 1). Washings were performed with phosphate buffered saline (PBS)/Tween20 pH 7.6. Reactions were revealed by Horseradish Peroxidase (HRP) - 3,3'-diaminobenzidine (DAB) Detection Kit (UCS Diagnostic, Rome, Italy). VDR expression was evaluated by counting the number of positive myonuclei (out of a total of 500 in randomly selected regions).

**Table 1:** List of primary antibodies.

Antibody	Dilution	Retrieval	Clone
Slow-myosin	1:100	EDTA citrate pH 7.8	NOQ7.5.4D, Abcam
Fast-myosin	1:100	EDTA citrate pH 8.0	MY-32, Abcam
PAX-7	1:200	Citrate pH 6.0	ab55494, Abcam
VDR	1:100	Citrate pH 6.0	2F4, Novus Biologicals

### Transmission electron microscopy (TEM) 

Small fragments of muscle tissue from surgical specimens (1 mm^3^) were fixed in 4% paraformaldehyde and post-fixed in 2% osmium tetroxide. After washing with 0.1 M phosphate buffer, the sample was dehydrated by a series of incubations in 30%, 50% and 70% ethanol. Dehydration was continued by incubation steps in 95% ethanol, absolute ethanol and propylene oxide, after which samples were embedded in Epon (Agar Scientific, Stansted, Essex CM24 8GF United Kingdom). Eighty µm ultra-thin sections were mounted on copper grids and examined with a transmission electron microscope (Model JEM1400, JEOL).

### Analysis of VDR polymorphisms 

#### Isolation of DNA

Genomic DNA was isolated from muscle section of paraffin-embedded tissue using automatic extraction *BioRobot EZ1 Advanced XL* (*QIAGEN*) in association with *EZ1 DNA Investigator kit* (*QIAGEN*) according to standard and pre-treatment procedure. DNA concentration and quality were assessed by *NanoDrop ND-1000* Spectrophotometer.

#### Genotyping of the *VDR* gene

The *Cdx2* (rs11568820), *FokI* (rs2228570), and *TaqI* (rs731236) polymorphisms of the *VDR* gene have been genotyped using the following predesigned *TaqMan SNP probe Genotyping Assays* (*Applied Biosystem*): Assay ID: C_2880808_10 (*Cdx2*), Assay ID: C_12060045_20 (*FokI*) and Assay ID: C_2404008_10 (*TaqI*). The reaction was performed by *ABI 7500 Fast Real-Time PCR System* (*Applied Biosystem*) according to standard protocol (95°C for 10 minutes and 40 cycles of 92°C for 15 second and 60°C for 1 minute). Genotyping analysis was performed by *SDS* (*Sequence Detection System*) Software that uses the fluorescence measurements made during the plate read to plot fluorescence (Rn) values based on the signals from each well. The plotted fluorescence signals indicate which alleles are in each sample (Biosystem 2006). Control samples representing all three possible genotypes (sample previously confirmed by direct sequencing as heterozygous, wild-type homozygous and/or variant homozygous) and a negative control were included in each reaction.

**Figure 1.**
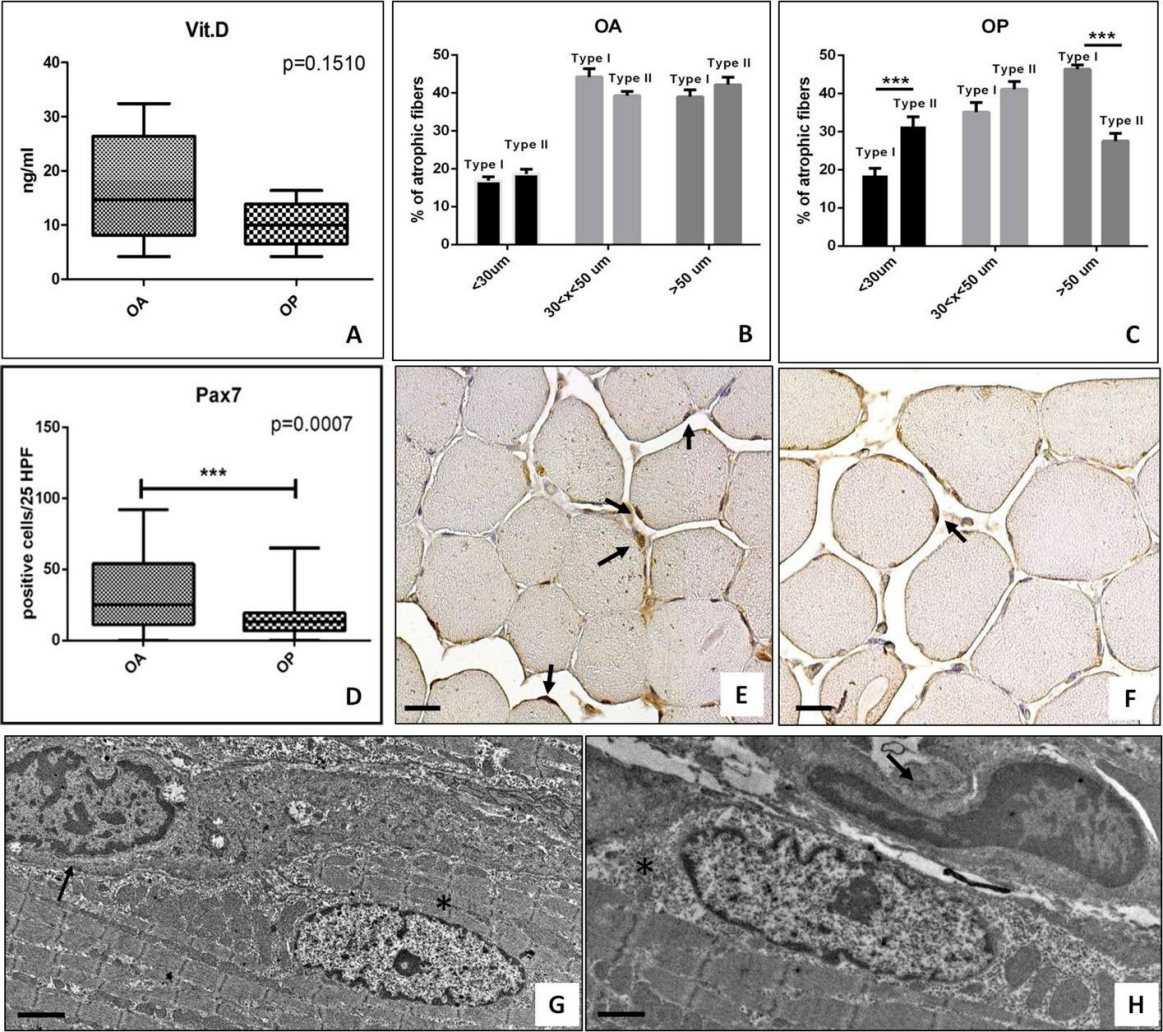
Vitamin D serum level and muscle quality. (A) Graph shows mean value of vitamin D serum concentration in OA e OP patients. (B) Graph displays the percentage of atrophic fibers in OA patients. (C) Graph displays the percentage of atrophic fibers in OP patients. (D) Graph shows higher number of Pax7 positive satellite cells in OA as compared OP patients. (E) Representative image of anti-Pax7 immunoreaction of a muscle biopsies of an OA patient (scale bar represents 50µm). Arrows indicate several Pax7 positive myonuclei. (F) Representative image of anti-Pax7 immunoreaction of a muscle biopsies of an OP patient (scale bar represents 50µm) Arrow indicates a Pax7 positive cell. (G) Electron micrograph displays a well conserved satellite cell (arrow) net to myonucleus (asterisk) in a muscle biopsy of an OA patient (scale bar represents 2µm). H) Electron micrograph shows a satellite cells in degeneration (arrow) next to myonucleus (asterisk) in a muscle biopsy of an OP patient (scale bar represents 2µm).

**Table 2:** Baseline characteristics of AO and OP patients.

	OA	OP

Age	74.42±1,25	70.90±1,46
	
Body Mass Index	25.84 ± 0.85	23.33 ± 3.21
	
T score L1-L4	-1.4 ± 1.12	-2.4 ± 1.08
	
Femoral neck T score	-1.2 ± 0.99	-2.99 ± 1.12
	
Kellgren-Lawrence grade	3-4	1-2
	
25(OH)D (ng/ml)	16.90±2,75	10.12±1,41

### Statistical Analysis

Difference of the 25(OH)D serum concentration, percentage of atrophic fibers, number of pax7 satellite cells and VDR expression between OA and OP groups was performed by Mann-Whitney test (p < 0.0005) (GraphPad Prism 5 Software, La Jolla, CA, USA).

The Hardy-Weinberg equilibrium was verified for all single nucleotide polymorphisms (SNPs) by the Pearson χ2 test. Differences in alleles and genotypes frequencies between groups of patients (OP and OA) were evaluated by the Fisher's Exact test. Genetic statistical analyses were performed by online *VassarStats Statistical Software*. Two-tailed P values less than 0.05 were considered statistically significant.

## RESULTS

### Clinical evaluation

The OP group included 44 patients with fragility hip fracture, T-score ≤-2.5 SD and K - L score from 0 to 1 (Table.2). The OA group included 55 patients with radiographic evidence of hip OA with a K - L score 3 or 4 and T-score ≥-2.5 SD (Table 2). There was no discrepancy for age, sex and comorbidities in the two groups. Specifically, no patient showed oncological, genetical or neurological diseases, whereas more of 80% of patients were affected by hypertension. Body Mass Index (BMI) mean value of OA patients was significantly higher than BMI mean value of OP group (mean value 25.84 ± 0.85 vs 23.33 ± 3.21, P <0.001) (Table 2). These data confirmed the frequently overweight condition of OA patients. No significant difference was observed for the serum concentration of (25(OH)D) (OA 16.90±2,75 ng/ml OP 10.12±1,41 ng/ml) (Table 2 and Fig. 1A). Also, in the OA group we observed a great variability in the value of 25(OH)D serum concentration respect to OP patients. However, more of 90% of patients (both OA and OP) were characterized by an hypovitaminosis condition. Indeed, hypovitaminosis D is defined as a serum 25(OH)D level of less than 20 ng/mL. Finally, no significant differences were observed comparing the 25(OH)D serum concentration with the patient’s age (data not shown).

### Morphometric examination

Slow myosin antibody and fast myosin antibody stains allowed us to discriminate type I and type II fibers, respectively (Fig. 1B, C). The morphometric analysis of muscle fibers in OA patients showed about 35.00% of atrophic fibers with a diameter of less than 30?μm (type I 16.81±1.04% e type II 18.60±1.51%) (Fig. 1B). In OP group, we observed about 50.00% of atrophic fibers with a statistically significant prevalence of type II fibers (type I 18.53±1.83% e type II 31.37±2.54%, p<0.0001) (Fig. 1C).

### Satellite cells analysis

Immunohistochemistry results of Pax7 expression were quantified by counting the number of positive satellite cells in 25 High Power Field (HPF) of randomly selected regions.

Our results showed a decrease of the number of Pax7 positive satellite cells in OP patients as compared to OA group (Fig. 1D, E, F). In particular, Mann-Whitney test displayed a greater significant difference for the number of Pax7 positive satellite cells (OA 32.40±3.70, OP 15.75±2.85 p=0.0075).

TEM analysis was performed to characterize satellite cells niches and their cell syncytium (Fig. 1). In OA muscle biopsies, we found well conserved satellite cells strongly associated among them or fused to form a syncytium (Fig. 1G). Conversely, in OP patients we observed numerous atrophic fibers and rare satellite cells with obvious mark of degeneration (Fig. 1H).

### VDR evaluation by immunohistochemistry

**Figure 2.**
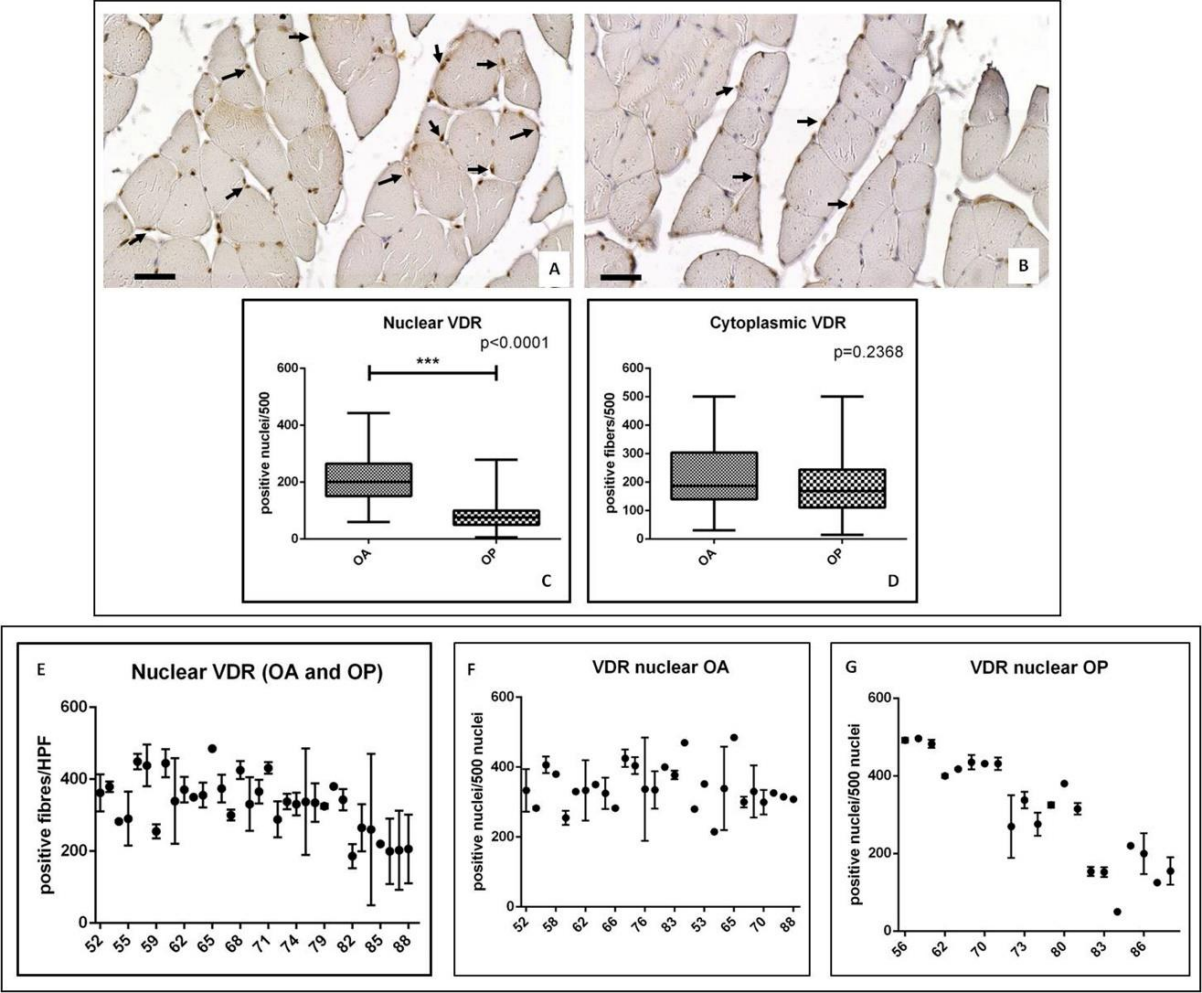
VDR expression in muscle biopsies of OA and OP patients. (A) Representative image of anti-VDR immunoreaction of a muscle biopsies of an AO patient (scale bar represents 50µm). Arrows indicate numerous VDR positive myonuclei. (B) Representative image of anti-VDR immunoreaction of a muscle biopsies of an OP patient (scale bar represents 50µm). Arrows indicate rare VDR positive myonuclei. (C) Graph shows higher number of VDR positive myonuclei in OA patients as compared to OP. (D) There is no significant difference between cytoplasmic expression of VDR in OA and OP patients. (E) Graph displays correlation between VDR nuclear expression and the age of the patients. (F) Graph shows correlation between VDR nuclear expression and the age of OA patients. Graph displays correlation between VDR nuclear expression and the age of OP patients.

Immunohistochemistry results of VDR were quantified by counting both the number of positive myonuclei and the number of positive fibers (out of a total of 500 in randomly selected regions) (Fig. 2A, B).

Mann-Whitney test displayed a significant difference in the nuclear expressions of VDR in OA group as compared to OP patients (OA 212.10±11.85, OP 84.73±7.29, p<0.0001) (Fig. 2C). Conversely, no significant difference was observed for cytoplasmic expression of VDR (OA 232.00±16.31, OP 190.30 ± 17.64, p=0.2368) (Fig. 2D).

In order to investigate the effect of aging on VDR activation, we plot the number of positive nuclei with the patient’s age (Fig. 2E, F, G). In line with results obtained by Ferrari et al., in the univariate analyses we found that increased age was associated with decreased of nuclear VDR (Fig. 2E). Noteworthy, analyzing OP and OA patients separately, we found very different “age effect” on VDR activation (positive nuclei) (Fig. 2 F, G). In particular, the nuclear translocation of VDR appeared age independent in OA group (Fig. 2F) and strictly influenced by age in OP patients (Fig. 2G). On note, under 60 OP patients showed a higher number of VDR positive myonuclei respect to under 60 OA patients.

### VDR polymorphisms analysis

In order to clarify the role of nucleotide variants in the *VDR* gene on its expression, we genotyped three major polymorphisms known to be involved in muscle and bone diseases in our Italian cohort of OP (n=44) and OA (n=55) patients. *Cdx2* polymorphism consists in G-A substitution in the *VDR* promoter region affecting the *VDR* expression level [19, 26]; *FokI* (C-T substitution) located in the first of two potential translation initiation sites in *VDR* exon 2 [19, 34] has a role in the regulation of transcription of vitamin D-dependent genes and *TaqI* polymorphism, a T-C synonymous substitution in *VDR* Exon 9 and its function is associated with increased stability of mRNA. [20, 35].

For *Cdx2* polymorphism, the alleles frequencies were G=83% and A=17% in OP patients (genotypes GG n=31, GA n=11, AA n=2) and G=65% and A=35% in OA patients (genotypes GG n=20, GA n=31, AA n=4). The alleles frequencies for *FokI* polymorphism were C=60% and T=40% in OP patients (genotypes CC n=14, CT n=25, TT n=5) and C=66% and T=34% in OA patients (genotypes CC n=24, CT n=25, TT n=6). Finally, for *TaqI* polymorphism the alleles frequencies were T=60% and C=40% in OP group (genotypes TT n=15, TC n=23, CC n=6) and T=52% and C=48% in OA group (genotypes TT n=14, TC n=29, CC n=12). Deviations from Hardy-Weinberg equilibrium were not observed for all three SNPs (*p *> 0.05) (Table 3).

**Figure 3.**
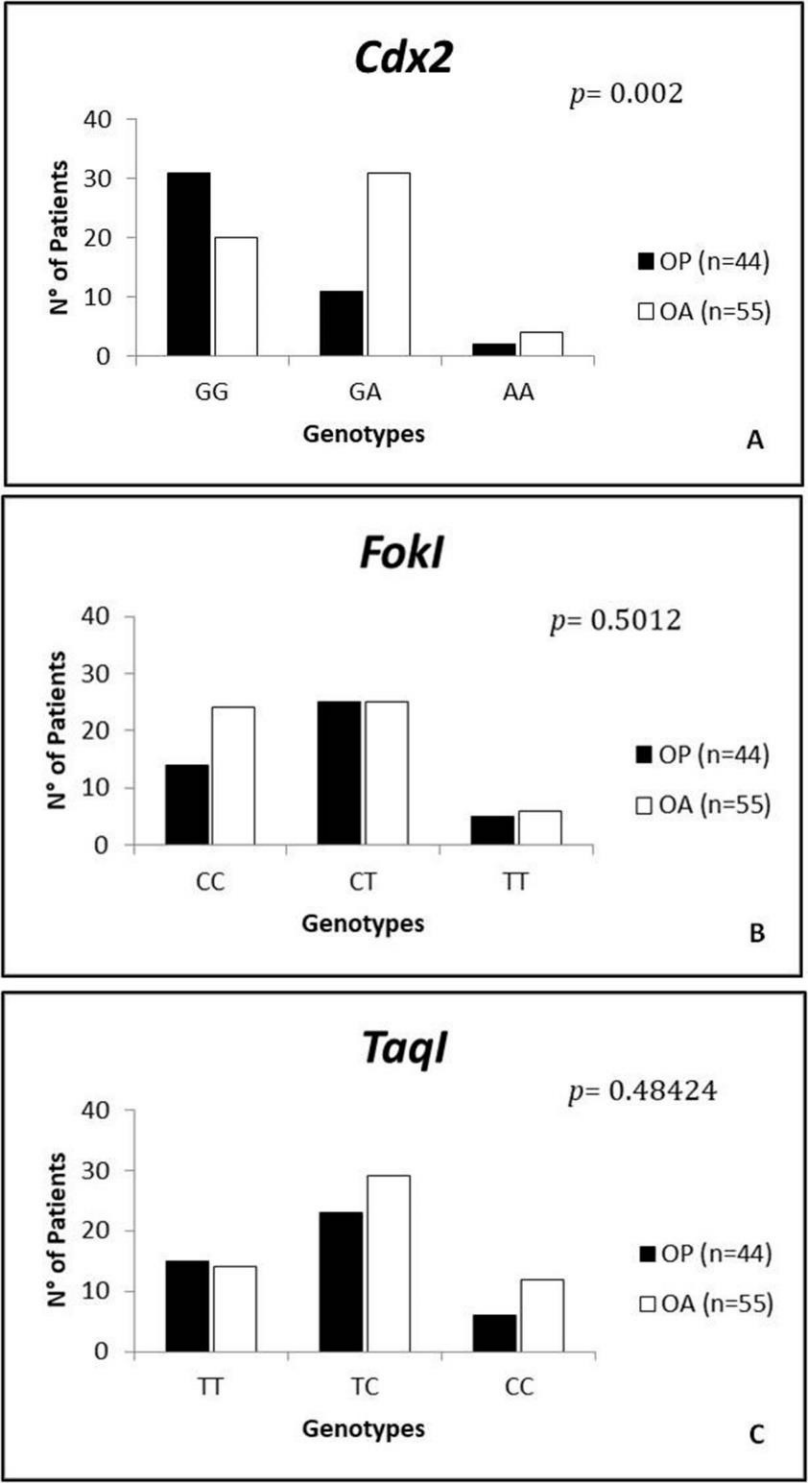
Genotypes distribution of the Cdx2, FokI and TaqI polymorphisms between OP and OA groups. (A) Fisher’s Exact test analysis (online VassarStats Statistical Software) showed a significant difference in genotype distribution for Cdx2 polymorphism between the OP and OA groups (p= 0.002). (B, C) There was not a significant difference in in genotype distribution for FokI and TaqI (0.50 and 0.48 respectively) polymorphisms in OP and OA patients (B-C). P-value was calculated using Fisher Exact Probability Test for a two-rows by three-columns contingency table (*p*< 0.05).

**Table 3:**
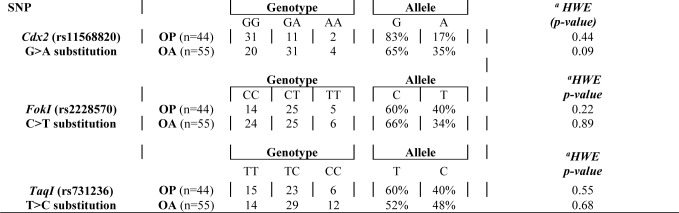
Genotypes and Alleles frequencies of VDR Gene Polymorphisms in OP and OA patients. Deviations from Hardy-Weinberg equilibrium for the three SNPs were not observed for all three SNPs: *Cdx2, FokI* and *TaqI (p > 0.05).*

a HWE: Hardy-Weinberg Equilibrium

Fisher's Exact test showed a significant difference in the genotype distribution of the *Cdx2* polymorphism between OP (n=44; GG n=31, GA n=11, AA n=2) and OA (n=55; GG n=20, GA n=31, AA n=4) groups (*p*=0,002) (Fig. 3A). In particular, the frequency of the A allele of the Cdx-2 polymorphism of the *VDR* gene was significantly higher (35%) in the OA group compared to OP group (17%). A higher percentage of OP patients was found with GG genotype (n=31) compared to OA (n=20) (Fig. 3A). In contrast, the GA genotype was more prevalent in the OA group (n=31) compared to OP (n=11) (Fig. 3A). Conversely, no significant differences in genotype distribution was observed relatively to *FokI* (*p*=0,50) and *TaqI* (*p*=0,48) polymorphisms in both OP (n=44; CC n=14, CT n=25, TT n=5 for *FokI* and TT n=15, TC n=23, CC n=6 for *TaqI*) and OA (n=55; CC n=24, CT n=25, TT n=6 for *FokI* and TT n=14, TC n=29, CC n=12 for *TaqI*) patients (Fig. 3B, C).

**Figure 4.**
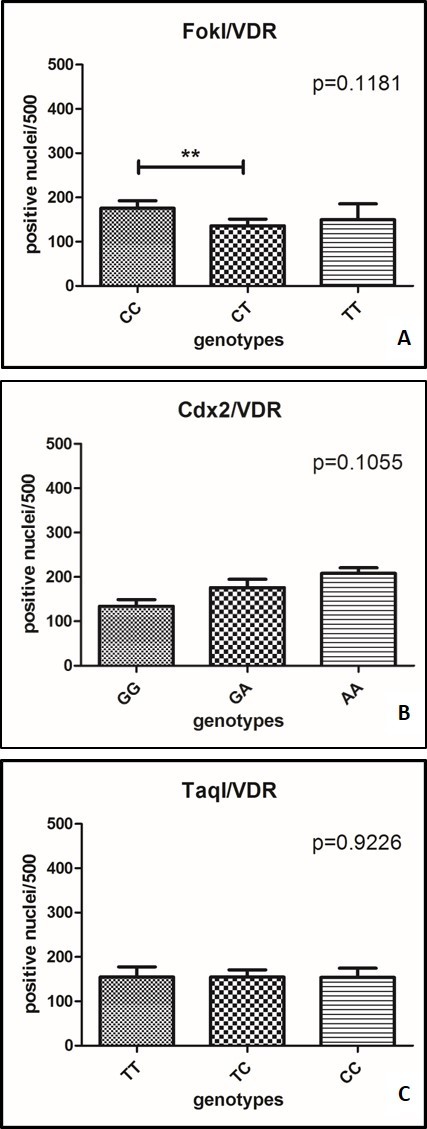
Comparison between *FokI*, *Cdx2* and *TaqI* polymorphisms and the number of positive myonuclei. (A) The comparison between *FokI* polymorphism and the number of positive myonuclei showed a significant difference in patients with CC genotype respect to patients with CT genotype (*p*=0.0044). (B) No significant difference in the nuclear expression of VDR was observed in *Cdx2*. (C) No significant difference in the nuclear expression of VDR was observed in *TaqI.*

To understand the possibility that the significant difference in the nuclear expressions of VDR in OA group compared to OP patients is attributable to a difference in the nucleotide variants in the *VDR* gene, we investigate both the three main polymorphisms in the *VDR* gene and the number of positive myonuclei in both OP and OA groups (Fig. 4).

The comparison between *FokI* polymorphism and the number of positive myonuclei showed a significant difference in patients with CC genotype respect to patients with CT genotype (*p*=0.0044) (Fig. 4A). On the other hand, no significant difference in the nuclear expression of VDR was observed in *Cdx2* (*p*=0.1055) (Fig. 4B) and *TaqI* polymorphisms (*p*=0.9226) (Fig. 4C).

**Figure 5.**
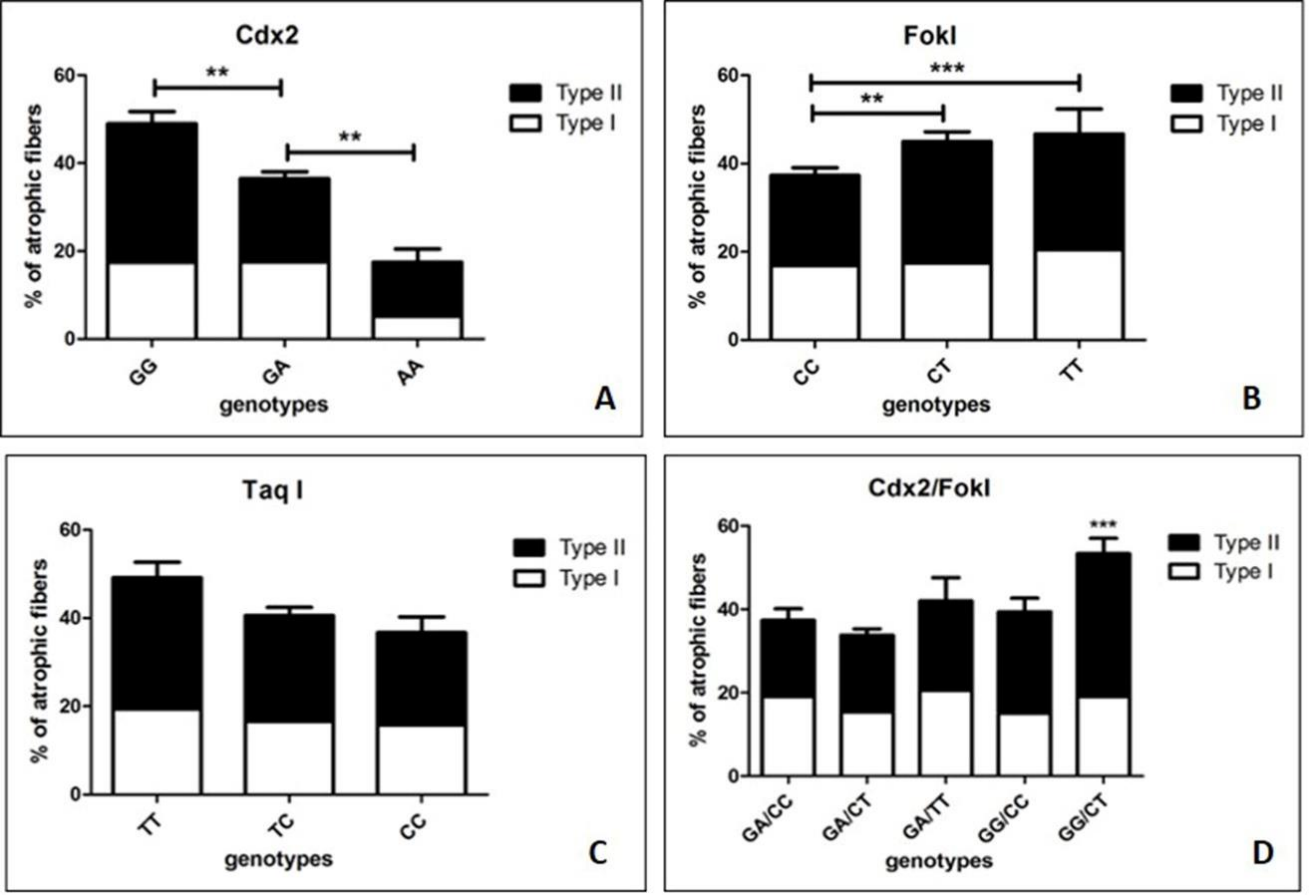
Comparison between *FokI, Cdx2* and *TaqI* polymorphisms and the percentage of positive myonuclei. (A) The comparison between *Cdx2* genotypes and muscle atrophy displays a significant group effect (GG =50%; GA= 38%; AA=18%; p=0,0004). Mann-Whitney test shows significant higher percentage of muscle atrophy (Type I = 17% vs Type II = 33%) in patients with GG genotype respect to patients with GA genotype (Type I = 17% vs Type II = 19%) (p=0,0035) and in patients with GA (Type I = 17% vs Type II = 19%) respect to patients with AA (Type I = 5% vs Type II = 13%) genotypes (p=0.0004). (B) The comparison between genotypes of *FokI* polymorphisms and muscle atrophy (CC =38%; CT= 45%; TT=46%) did not show a significant group effect (p=0,1080). Mann-Whitney test shows significant higher percentage of muscle atrophy in patients with CT genotype (Type I = 17% vs Type II = 28%) compared to patients with CC genotype (Type I = 17% vs Type II = 20%) (p= 0,0440). (C) The comparison between genotypes of *TaqI* polymorphisms and muscle atrophy (TT=50%; TC= 40%; CC=37%) did not reveal a significant group effect (p=0,1282). (D) Patients with GG/CT and GG/CC genotypes showed a significant higher percentage of type II atrophic fibers (Type II =35% and 25% respectively) respect to other genotypes combination (Type I =18% and 15% respectively).

In order to circumstantiate the effect of *VDR* polymorphisms on muscle health, we also evaluated the putative relationship among *VDR* polymorphisms and percentage of atrophic fibers in both groups of OP and OA patients. The comparison between *Cdx2* genotypes and muscle atrophy, evaluated in terms of percentage of atrophic muscle fibers (both type I and II), displayed a significant group effect (GG =50%; GA= 38%; AA=18%) (*p*=0,0004) (Fig. 5). Mann-Whitney test showed significant higher percentage of muscle atrophy (Type I = 17% vs Type II = 33%) in patients with GG genotype respect to patients with GA genotype (Type I = 17% vs Type II = 19%) (*p*=0,0035) (Fig. 5A). In addition, we also observed a significant difference, in term of muscle atrophy, between patients with GA (Type I = 17% vs Type II = 19%) and AA (Type I = 5% vs Type II = 13%) genotypes (*p*=0.0004) (Fig. 5A).

The comparison between genotypes of *FokI* polymorphism and muscle atrophy (CC =38%; CT= 45%; TT=46%) did not show a significant group effect (*p*=0,1080) (Fig. 5B). Conversely, Mann-Whitney test showed significant higher percentage of muscle atrophy in patients with CT genotype (Type I = 17% vs Type II = 28%) compared to patients with CC genotype (Type I = 17% vs Type II = 20%) (*p*= 0,0440) (Fig. 5B).

On the other hand, the comparison between genotypes of *TaqI* polymorphism and muscle atrophy (TT=50%; TC= 40%; CC=37%) did not reveal a significant group effect (*p*=0,1282) (Fig. 5C). Mann-Whitney test did not display significant differences among the analyzed groups (Fig. 5C).

Finally, we also analyzed the combination of the different genotypes of the two functional polymorphisms of the *VDR* genes, *Cdx2* and *FokI*, in relationship with percentage of atrophic fibers evaluated in both OP and OA groups (Fig. 5D). Our results clearly demonstrated the increase of muscle atrophy (>50%) in patients characterized by GG/CT genotypes (associated to lower biological activity of VDR) respect to the other combinations (Fig. 5D). Moreover, patients with GG/CT and GG/CC genotypes showed a significant higher percentage of type II atrophic fibers (Type II =35% and 25% respectively) respect to other genotypes combination (Type I =18% and 15% respectively) (Fig. 5D).

### DISCUSSIONS

It is known that Vitamin D plays an essential role in skeletal muscle homeostasis, but its precise physiological function and relevance to normal muscle physiology is not well understood [17]. In this context, Bischoff-Ferrari et al. reported the expression of VDR in muscle tissues demonstrating that older age was significantly associated with decreased VDR expression, independent of biopsy location and serum 25(OH)D levels [18]. Starting from these evidences, in this study we investigated the relationship among muscle quality, evaluated in term of percentage of atrophic fibers, VDR nuclear expression and the main *VDR* polymorphisms associated with OP or OA namely *Cdx2*, *TaqI* and *FokI*. To this end, we collected biopsies of *vastus lateralis* from 99 patients who underwent hip surgery: 44 OP patients and 55 OA patients. Morphometrical examination showed a delay of the onset of sarcopenia in OA patients respect to OP. This data is also supported by the consistent storage of Pax7 positive satellite cells that we found in muscle tissue of OA patients. Satellite cells play an indispensable role in muscle regeneration. The self-renewing proliferation of these cells not only maintains the stem cell population but also provides numerous myogenic cells, which proliferate, differentiate, fuse, and lead to new myofiber formation and reconstitution of a functional contractile apparatus [36]. The loss of satellite cells, and/or their degeneration, could reflect the alteration of muscle metabolism that occurs in patients affect by osteoporosis. In this context, we recently showed that muscle tissues from OP patients were characterized by the imbalance between myostatin and bone morphogenetics proteins (BMPs) pathways [37,38,39,40]. VDR was evaluated both at cytoplasmic and nuclear level by immune-histochemistry. Our results revealed a higher number of VDR positive nuclei in OA patients respect to OP. Conversely, no significant difference was observed respect to VDR cytoplasmic expression. It is important to note that these differences are not influenced by the serum level of 25(OH)D. Indeed, both our experimental groups were characterized by a condition of hypovitaminosis with very similar mean values of 25(OH)D serum concentration. On note, OA group was characterized by patients with a wide range of 25(OH)D values (min 4,20 - max 32,40 ng/ml).

In line with the results of Bischoff-Ferrari et al., we observed a general decrease of VDR positive myonuclei with the age considering both OA and OP patients. Noteworthy, analyzing the experimental groups individually, for the first time, we reported that aging differently affects the VDR activation in OA and OP patients. In particular, while in OP patients we observed a significant reduction of VDR positive myonuclei with age, no “age effect” was observed in OA patients. The frequent activation of VDR could explain the lower number of atrophic fiber that we observed in OA patients respect to OP. Indeed, Braga et al. demonstrated that the activation of VDR in satellite cells induce the expression of known markers of muscle regeneration such as MyoD, myogenin, insuline-like growth factor-1 (IGF-1) and BMPs [40]. These evidences are also supported by our recent studies in which we demonstrated the high expression of BMP-2/4/7 and myogenin in muscle tissues of OA patients.

In order to better elucidate the role of VDR on muscle homeostasis and therefore explain the different pattern of VDR activation in OA and OP patients, we analyzed the *Cdx2*, *TaqI* and *FokI* VDR polymorphisms. The genotype distribution of the *Cdx2* polymorphism showed a significant difference between OP and OA groups. In particular, we observed a higher frequency of A (35%) allele in the OA group compared to OP group (17%). Moreover, in OA patients we observed a significant increase of VDR positive myonuclei and the lower number of atrophic fiber compared to OP patients. No significant differences have been observed in genotype distribution of *FokI* and *TaqI* polymorphisms between OP and OA groups.

To investigate the influence of VDR polymorphisms on muscle quality, we studied the putative association between their genotypic distribution and the percentage of atrophic fibers in both OA and OP patients.

In this study, even if in a small sample size, we found that the G allele of *Cdx2* polymorphism of *VDR* gene was significant associated with higher percentage of the atrophic type II fibers. The putative functional role of this allele is to decrease the binding activity of the Cdx2 protein on the *VDR* promoter thus reducing the transcription level of the *VDR* gene itself [25]. This finding suggests that the G allele could be responsible for the lower activation of VDR in satellite cells leading to reduced expression of gene implicated in muscle regeneration and consequent fiber atrophy in OA and OP groups. The second *VDR* polymorphism characterized is *FokI*, which is known to affect the translational start site of the *VDR* gene. OP and OA patients carrying the CT genotype showed more atrophic fibers compared to patients with CC genotype. These results are in line with literature data demonstrating that individuals with the T allele synthesize the full-length VDR protein (isoform T, 427 amino acids), while individuals with the C allele synthesize a slightly truncated version of the VDR protein (isoform C, 424 aminoacids). The truncated C isoform of the VDR proteins displays a higher transcriptional activation of reporter genes *in vitro* and interacts more efficiently with the key transcription factor TFIIB, than the longer T isoform [42]. It is important to note that both OA and OP patients with the combination of the GG/CT genotypes showed a significant increase in the number of type II atrophic fibers respect to the other *VDR* genotype combinations [2]. Another important aspect was the correlation between the number of VDR-positive myonuclei and *VDR *polymorphisms genotypes. We observed a significant correlation between the CC *FokI* genotype, which activates the VDR transcription, and the nuclear localization of the VDR protein. On the basis of this observation, it is possible to speculate that *FokI* polymorphism could also promote the translocation of the VDR protein from cytoplasm to cell nucleus. Further functional studies are needed to verify this novel hypothesis which could provide additional information about the role of VDR in muscle-skeletal pathway. To the best of our knowledge, this is the first report demonstrating this association, however, given the small sample size, genotyping of these SNPs is recommended to be carried out in different populations with more samples. Overall, our data highlight the prominence of VDR activity and genetic variability on muscle aging and sarcopenia typical of OA and OP patients.

### Conclusions

In the era of personalized medicine, the identification of molecules/genes involved in aging of musculoskeletal apparatus represent one of the most important scientific research fields.

In this context, here we showed as the activation of VDR (nuclear translocation) is strictly associated with the percentage of atrophic muscle fibers. These preliminary evidences, if confirmed in larger cohort of samples, will provide new insights in the pathogenesis and age-related muscle disorders based on the genotyping of the *VDR* gene.
